# Supplementation of polyunsaturated fatty acids, magnesium and zinc in children seeking medical advice for attention-deficit/hyperactivity problems - an observational cohort study

**DOI:** 10.1186/1476-511X-9-105

**Published:** 2010-09-24

**Authors:** Michael Huss, Andreas Völp, Manuela Stauss-Grabo

**Affiliations:** 1 Department for Child and Adolescent Psychiatry, Johannes Gutenberg-University, Mainz, Langenbeckstr. 1; 55131 Mainz, Germany; 2 Psy Consult Scientific Services, Fuchstanzstr. 107, 60489 Frankfurt/Main, Germany; 3 Engelhard Arzneimittel GmbH & Co.KG, Herzbergstrasse 3, 61138 Niederdorfelden, Germany

## Abstract

**Background:**

Polyunsaturated fatty acids are essential nutrients for humans. They are structural and functional components of cell membranes and pre-stages of the hormonally and immunologically active eicosanoids. Recent discoveries have shown that the long-chained omega-3 fatty acids eicosapentaenoic acid (EPA) and docosahexaenoic acid (DHA) also play an important role in the central nervous system. They are essential for normal brain functioning including attention and other neuropsychological skills.

**Materials and methods:**

In our large observational study we monitored 810 children from 5 to 12 years of age referred for medical help and recommended for consuming polyunsaturated fatty acids (PUFA) in combination with zinc and magnesium by a physician over a period of at least 3 months. The food supplement ESPRICO^® ^(further on referred to as the food supplement) is developed on the basis of current nutritional science and containing a combination of omega-3 and omega-6 fatty acids as well as magnesium and zinc. Study objective was to evaluate the nutritional effects of the PUFA-zinc-magnesium combination on symptoms of attention deficit, impulsivity, and hyperactivity as well as on emotional problems and sleep related parameters. Assessment was performed by internationally standardised evaluation scales, i.e. SNAP-IV and SDQ. Tolerance (adverse events) and acceptance (compliance) of the dietary therapy were documented.

**Results:**

After 12 weeks of consumption of a combination of omega-3 and omega-6 fatty acids as well as magnesium and zinc most subjects showed a considerable reduction in symptoms of attention deficit and hyperactivity/impulsivity assessed by SNAP-IV. Further, the assessment by SDQ revealed fewer emotional problems at the end of the study period compared to baseline and also sleeping disorders. Mainly problems to fall asleep, decreased during the 12 week nutritional therapy. Regarding safety, no serious adverse events occurred. A total of 16 adverse events with a possible causal relationship to the study medication were reported by 14 children (1.7%) and only 5.2% of the children discontinued the study due to acceptance problems. Continuation of consumption of the food supplement was recommended by the paediatricians for 61.1% of the children.

**Conclusion:**

Our results suggest a beneficial effect of a combination of omega-3 and omega-6 fatty acids as well as magnesium and zinc consumption on attentional, behavioural, and emotional problems of children and adolescents. Thus, considering the behavioural benefit in combination with the low risk due to a good safety profile, the dietary supplementation with PUFA in combination with zinc and magnesium can be recommended.

## Background

Fish is brain food, a popular opinion says. Due to its high amount of polyunsaturated fatty acids (PUFA), such as omega-3 and omega-6, which are essential for all mammals, fish consumption is regarded to be advantageous for brain development and function [1,2]. A possible reason is that phospholipids containing PUFA are integral parts of the neuronal cell membranes of the brain, and they might be important for facilitating the transmission of signals between neurons [3]. Furthermore, micronutrient intake, e.g. iron, magnesium and zinc, has been linked to the development, structure and function of the brain [4]. Consequently, an emerging literature deals with the relationship between nutrition, attention and executive functions in the development of children and adolescents [5,6].

Evidence indicates that PUFA may play an important role in the prevention and treatment of certain mental health disorders [7,8] like, for example, attention deficit hyperactivity disorder (ADHD). ADHD is one of the most common behavioural childhood disorders with prevalence rates of 5.29% worldwide [9] and 4.8% in Germany [10]. Several studies have been conducted, suggesting a link between omega-3/omega-6 fatty acids on the one hand and ADHD symptoms and learning difficulties on the other [11]. Trials with supplementation of a combination of two long-chained omega-3 acids, i.e. eicosapentaenoic acid (EPA) and docosahexaenoic acid (DHA), in children with reading writing disorder (RWD), dyspraxia (i.e., motor coordination difficulties or developmental coordination disorder), and ADHD-related symptoms have reported significant symptom reductions [12-14]. In contrast, trials with DHA only have been less promising [15,16], suggesting that the presence of the EPA component might be important.

The role of micronutrients has been investigated as well. For example, a study of 48 children [17] suggests that low zinc levels are correlated with inattentiveness symptoms in children with ADHD. Another randomized, controlled trial of 608 children found that, over a 14-month period, micronutritient supplementation improved attention-concentration symptoms in school children [18]. Nogovitsina and Levitina [19] identified decreased plasma and erythrocyte magnesium levels and decreases in Mg(2+)-ATPase activity in children with ADHD.

However, the role of specific diets or dietary nutrients in the prevention and management of ADHD is still questioned [20,21]. A recent review indicates that the evidence is best for zinc [22]. Moreover, a mono-causal role for nutrient deficiencies in children with ADHD or a role for a specific diet in the treatment of ADHD seems to be unlikely. So far, in ADHD guidelines [23] multimodal treatment is recommended including psycho education, psychosocial interventions, psychotherapy, and pharmacotherapy. Recommendations for dietary nutrients have not yet been implemented. However, many parents follow general nutritional recommendations for the intake of PUFAs and nutrients in order to improve their children's behavioural problems [24].

Our large observational study focuses on children whose parents judged them to be prone to inattentiveness and hyperactivity and therefore consulted their paediatricians. The paediatricians recommended treatment with ESPRICO^®^, a chewable capsule which was developed on the basis of current nutritional science containing a combination of omega-3 and omega-6 fatty acids as well as magnesium and zinc, to support attention. The percentage of the EPA in relation to DHA is intentionally high, because studies indicate that especially EPA can effectively improve perception, attention, memory and mood [13,25-27]. To optimize the benefit for children with attention problems, the food supplement also contains the omega-6 fatty acid gamma-linolenic acid (GLA) and the minerals magnesium and zinc.

By monitoring the children over a period of at least 12 weeks, we evaluated the effects of this dietary measure. In addition, the influence of ESPRICO^® ^on impulsivity, hyperactivity, emotional problems, and sleep related symptoms, as well as the dietary supplement's safety and acceptance were investigated.

## Methods

### Study objective

The primary objective was to evaluate the nutritional effects of PUFA in combination with magnesium and zinc in children on the core symptoms of ADHD (inattention, impulsivity/hyperactivity) in a primary care setting. Following a dimensional construct of ADHD, the primary outcome was assessed dimensionally using standard ADHD scales. In line with the dimensional approach, we did not define severity cut-offs for the ADHD-scale nor did we define a-priori the existence of a formal ADHD diagnosis. The secondary objective was to investigate effects on emotional and behavioural problems as well as on sleep related disturbances in children who were referred for such problems. Finally profiles for tolerability, safety, and compliance were investigated.

### Study design and population

This was an observational, longitudinal study investigating possible effects of a dietary supplement containing a combination of omega-3 and omega-6 fatty acids as well as magnesium and zinc. The intended naturalistic study population was a sufficient large sample of children in primary care settings referred for attentional and behavioural problems in the age range of 5 to 12 years. Due to the strictly observational character of the study, no control group was included. Only children for whom the food supplement was indicated according to the clinical judgment of the paediatrician and who received the dietary supplement for the first time in their life were eligible for participation in the study. The consumption of the PUFA-magnesium-zinc combination was based on the clinical recommendation of the physician. Subject assessment was performed by interviews using internationally standardized questionnaires (see below) at baseline, after 2 to 4 weeks (interim interview) and at the end of the study period of 12 weeks (final interview). In cases of adverse events additional interim interviews could be conducted at any time. The final interview also included assessments of safety and acceptance using information provided by physicians and parents. Children suffering from supplement related allergies (e.g. fish and evening primrose oil and any ingredients of the food supplement) were excluded from the study.

### Treatment

ESPRICO^® ^is a product that contains a combination of omega-3 and omega-6 fatty acids as well as magnesium and zinc (Table 1). It is available as chewable capsules or sachets of suspension; the recommended daily dose of is 2 × 2 capsules or 1 sachet of suspension.

**Table 1:** Content of four capsules of ESPRICO^® ^(recommended daily dose).

Omega-3 EPA (eicosapentaenoic acid)	400 mg
Omega-3 DHA (docosahexaenoic acid)	40 mg

Omega-6 GLA (gamma-linolenic acid)	60 mg

Magnesium	80 mg (21% of RDA)

Zinc	5 mg (50% of RDA)

RDA: Recommended Daily Allowance.

### Assessment procedures

The assessments were obtained by the participating paediatricians during interviews with the children's parents. The data were documented using an electronic case report form. The following two standardized questionnaires were administered at baseline, after 2 to 4 weeks (interim interview) and after 12 weeks (final interview):

#### SNAP IV, assessment of attention deficit, hyperactivity/impulsivity

The SNAP-IV rating scale is a revised version of a questionnaire developed by Swanson, Nolan and Pelham (SNAP) used to measure attention deficit and hyperactivity symptoms [28] strictly following the international ADHD criteria. The scale contains a total of 30 questions (items). Because we wanted to investigate the nutritional effect of PUFA in combination with magnesium and zinc on inattention, impulsivity and hyperactivity, only the 18 items summarized by the subscales for attention deficit (item 1-9) and hyperactivity/impulsivity (item 11-19) were used in this investigation. These 18 items also cover the DSM-IV criteria for ADHD [29]. Each of the 18 questions had to be answered on a 4-point Likert-like scale ranging from 0 ("never or rarely") to 3 ("very often"). For both subscales separate item sum scores are calculated for each child, which then were added to obtain a total score with a (theoretical) range between 0 (absence of any symptoms) and 54 (all 18 items at maximum symptom frequency). The changes in average point scores (APS) between the three interviews (baseline, interim, final) are expressed as absolute and relative (percent) change from baseline.

According to the scale's standardization the majority of the population of children without ADHD falls within a total score range of 0 to 5. The maximum level is never reached in practice. A total score of 20 to 25 is considered as threshold for an ADHD diagnosis. Regarding the individual subscales, intra-individual APS item sum scores of 16 (subscale attention deficit; corresponding to a mean score of 1.78) and 13 (subscale hyperactivity/impulsivity; corresponding to a mean score of 1.44), respectively, are considered to be indicative of clinically relevant disorders. According to the authors of the scales these scores indicate the 95^th ^percentile of the SNAP-IV validation sample [28].

#### SDQ - Strengths and Difficulties Questionnaire

According to Goodman et al. the SDQ provides a general evaluation of emotional and behavioural problems [30,31]. The SDQ comprises a total of 25 questions (items) which are divided into five subscales entitled Emotional Problems, Behavioural Problems, Hyperactivity, Problems with Peers, and Pro-social Behaviour. To minimize compliance problems due to the length of assessment, 5 items of the SDQ were selected ("many worries, often seems worried", "often unhappy; down-hearted or tearful", "nervous or clingy in new situations; easily loses confidence", "many fears; easily scared"). These four items were taken from the subscale Emotional Problems whereas the fifths question "rather solitary, tends to play alone" was taken from the subscale Problems with Peers. Each question was evaluated on a three-point scale ranging from 0 ("not true"), over 1 ("somewhat true"), to 2 ("certainly true"). Furthermore, the four items taken from subscale Emotional Problems are summarized by an APS for which absolute and relative change from baseline are specified.

#### Evaluation of sleep

Based on information provided by parents and children throughout the study, the observing paediatricians documented potential sleep related problems, i.e. problems to fall asleep, to sleep through the night as well as impaired sleep quality. Answers were recorded as "yes" or "no", and for each item the rate of impairment at the end of the trial compared to impairment at baseline was determined.

### Data analysis and statistics

The observed data were analyzed descriptively, and all p values reported below are intended to be descriptive (values ≤0.05 are considered to be descriptively significant). Data management and statistical calculations were performed using Superbase for Windows 2001 database software and SPSS for Windows (version 15.0.1) statistical analysis software. Analyses were performed for the whole study population as well as for subgroups, i.e. different genders and age groups. All participating children were included in the analyses.

## Results

In total, 139 paediatric practices in Germany participated in the study recruiting 810 children and adolescents (see table 2), aged 4 to 15 years with nearly two-thirds (530; 65.4%) between 7 and 10 years. All of them were treated with the food supplement and were followed up over a period of at least 12 weeks. Reasons for recommendation are listed in table 3. The study did not interfere with any medical procedures including medication or other therapeutic interventions.

**Table 2:** Demographic characteristics of study population.

Study population (N)	810 (100%)
Male/female	579/231

Age (yr; median)	8.6

Weight (kg; mean)	31.3

Weight (kg; min-max)	13.8-87.2

Height (cm; mean)	134

Height (cm; min-max)	101-178

Existing diseases at baseline (N (%))	112 (13.8%)

Psychiatric disease at baseline (N (%))	41 (5.1%)

Concomitant medication at baseline (N (%))	59 (7.3%)

Dropouts therapy (N (%))	113 (14%)

**Table 3:** Reasons for treatment with ESPRICO^® ^(answers not mutual exclusive).

	N	%
Lack of concentration	746	92.1

Lack of attentiveness	681	84.1

Aggressive behavior	6	0.7

Agitation/hyperactivity	41	5.1

Enhancement of endurance and productivity	11	1.4

Sleeping disorders	3	0.4

Other indications	21	2.6

### Dropouts

During the study period 113 subjects (14%) discontinued the intake of ESPRICO^® ^prematurely. Reasons for dropout were mainly „absence of a positive effect" (50; 6.2%) and „lack of compliance" (42; 5.2%). Nine children (1.1%) did not take ESPRICO^® ^over the whole study period (12 weeks) because of adverse reactions. More than 96% of the subjects attended all three interviews: the baseline interview was performed in 810 children (100%), the interim interview in 781 children (96.4%) and final interview in 792 children (97.8%).

### Concomitant medication

At the start of the study 59 children (7.3%) received medical treatment. The most frequently listed medications were drugs for treatment of obstructive pulmonary diseases (N = 13; 1.6%), psychotropic drugs (N = 9; 1.1%) and thyroid therapeutics (N = 9; 1.1%). The psycho stimulants administered included methylphenidate (N = 7; 0.9%) and unspecified amphetamines (N = 1; 0.1%). Atomoxetine, the only non-stimulant medication approved for treatment of ADHD, was given to one child (N = 1; 0.1%). Four children (0.5%) were under treatment with the homeopathic complex (ingredients: chamomile dil., potassium phosphoricum, staphisagria, valeriana).

### Dietary therapy

During study participation the food supplement was administered for an average (± SD) period of 85 ± 19 days, with a maximum exposure of 156 days. 767 children (94.7%) received the recommended dose of 2 × 2 capsules per day (range: 2-8 capsules per day).

### Attention deficit

Initially, 308 children (38%) reached clinically relevant APS assessed by SNAP-IV in the area of attention deficit, i.e. an APS of 16 points or above. 227 (73.7%) of the children with initially elevated scores remained below this threshold at the final interview. Taking all subjects into account, APS for attention deficit showed a significant reduction from baseline to the end of the study by an average of 5.36 points (Table 4), corresponding to a relative improvement of 33.6% of the baseline score.

**Table 4:** Decrease in average point scores (APS) from baseline to the end of the study.

	N	Decrease in points (mean)	Standard deviation	95% Confidence interval	p*
Attention deficit (APS)	754	5.36	5.23	4.98-5.73	< 0,001

Hyperactivity/impulsivity (APS)	756	3.65	5.11	3.29-4.02	< 0,001

SNAP-IV (total score)	734	8.99	9.02	8.33-9.64	< 0,001

Emotional problems (APS)	781	0.25	0.52	0.21-0.29	< 0.001

* two-sided t-test.

**Figure 1:**
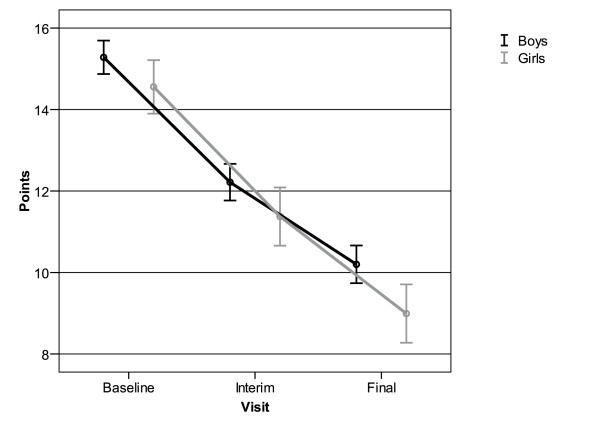
**Decrease in average point scores (APS) for attention deficit assessed by SNAP-IV for 551 boys and 225 girls**. Mean and 95% confidence interval.

The study sample included more than 70% boys whose attention deficit problems were slightly more pronounced than those of the participating girls. A gender-specific evaluation also revealed a clear reduction of the attention deficit symptoms as reflected by APS in boys and girls during the study period (Figure 1). The reduction was similar in both genders and averaged out at 5.1 ± 5.2 points for boys and 5.6 ± 5.4 points for girls (mean ± SD). Sub-analyses of different age groups showed no clinically significant differences in attention deficit reduction.

### Hyperactivity/Impulsivity

On the SNAP-IV Hyperactivity/Impulsivity subscale 320 children (39.5%) initially had an APS of 13 points or above and were thus classified to have a clinically relevant disorder in this particular area. At the end of the study, 202 of these children (63.1%) improved to an APS below the threshold of 13 points. Altogether, APS for hyperactivity/impulsivity decreased by at least 3.65 points over the study period (Table 4), corresponding to a relative improvement of 28.2% of the baseline score.

**Figure 2:**
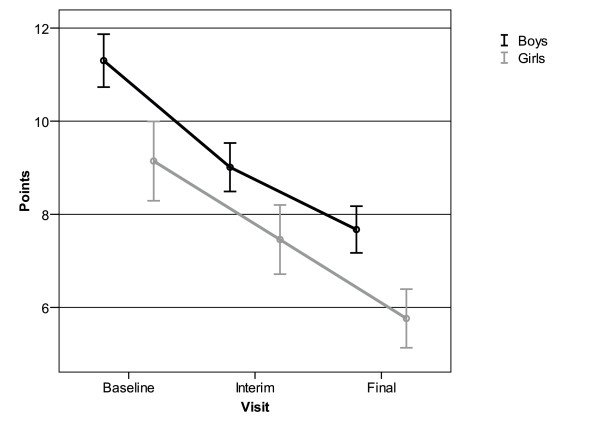
**Decrease in average point scores (APS) for hyperactivity/impulsivity assessed by SNAP-IV for 556 boys and 223 girls**. Mean and 95% confidence interval.

Results of gender-specific analyses showed a significantly higher impairment of boys in this area over the whole study period. The symptoms for impulsivity/hyperactivity as mirrored by APS were reduced by averages of 3.6 ± 5.2 points for boys and by 3.4 ± 4.8 points for girls (Figure 2). Thus, the symptoms were reduced to a similar degree in both genders in the course of the study.

**Figure 3:**
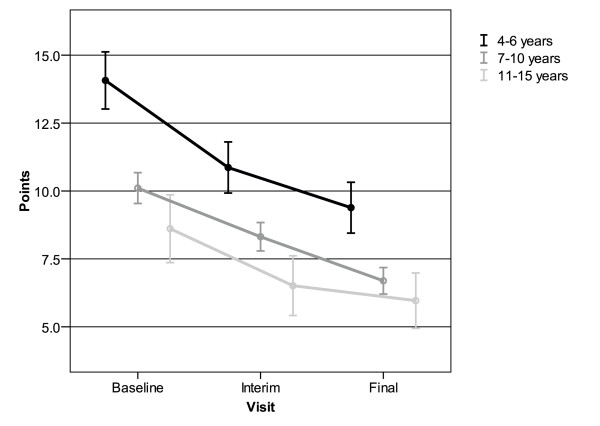
**Reduction of average point scores (APS) for hyperactivity/impulsivity assessed by SNAP-IV in children of different age groups**. Mean and 95% confidence interval (sample size, 4-6 yr: 155; 7-10 yr: 513; 11-15 yr: 111)

Sub-analyses of different age groups showed that children between 4 and 6 years of age (N = 155) presented with significantly higher pre-treatment hyperactivity/impulsivity scores than older children (Figure 3). With a mean value of 4.7 ± 5.9 points this age group also showed the most pronounced decrease in APS in this study, compared to decreases by 3.7 ± 4.9 points in 7-10 years old children and by 2.6 ± 4.4 points in 11-15 years old children.

### Emotional and behavioural problems

Between baseline and the final assessment the percentage of children who were described as loners (completely or partly true) decreased from 42.6% (345 of 810) to 32.2% (261 of 810).

Comparing baseline and final interview the summary score computed as the mean value of the four items from the SDQ subscale Emotional Problems decreased significantly by an average of 0.25 ± 0.52 points (Table 4), corresponding to a relative decrease by 28.1% of the baseline value.

Subgroup analyses of different age groups showed only slight differences in decrease of the summary score, but children between 4 and 6 years of age were generally less affected by emotional problems.

**Figure 4:**
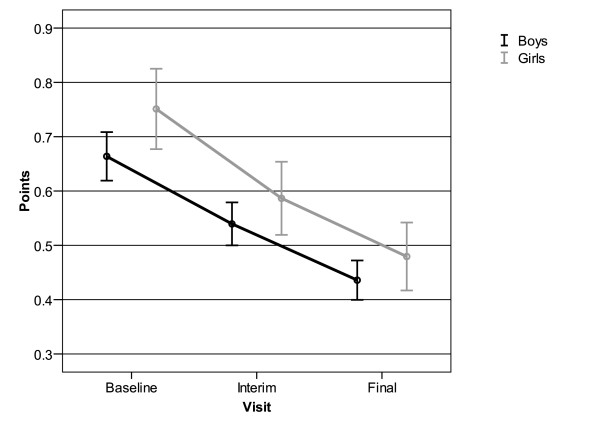
**Decrease in average point scores (APS) for emotional problems assessed by SDQ for 577 boys and 231 girls**. Mean and 95% confidence interval.

Gender-specific evaluation showed that emotional problems were more prevalent in girls. Reduction of symptoms as reflected by the summary score was similar for both genders with averages of 0.2 ± 0.5 points in boys and of 0.3 ± 0.6 points in girls (Figure 4).

### Sleep related problems

Information provided by parents and children during the interviews regarding falling asleep, sleeping through the night and sleep quality revealed that problems with falling asleep existed much more frequently than problems with sleeping through or reduced sleep quality. In the course of the observational period the percentage of children with sleep-related problems was reduced significantly in each of the three areas by more than 40% (Table 5).

**Table 5:** Number (%) of children with sleeping disorders.

	N	Baseline	Final interview	Decrease in % (95% Confidence interval)	p*
Problems to fall asleep	796	305 (38,3%)	182 (22,9%)	40.3 (28.1-50.6)	< 0,001

Problems to sleep through the night	797	148 (18,6%)	87 (10,9%)	41.2 (22.9-55.4)	< 0,001

Reduced sleep quality	784	186 (23,7%)	107 (13,6%)	42.4 (26.6-55.1)	< 0,001

* two-sided chi-square test.

Sub-analyses of different age groups showed only slight differences, with older children having more problems to fall asleep. Improvement of sleep related symptoms was similar in all age groups.

**Figure 5:**
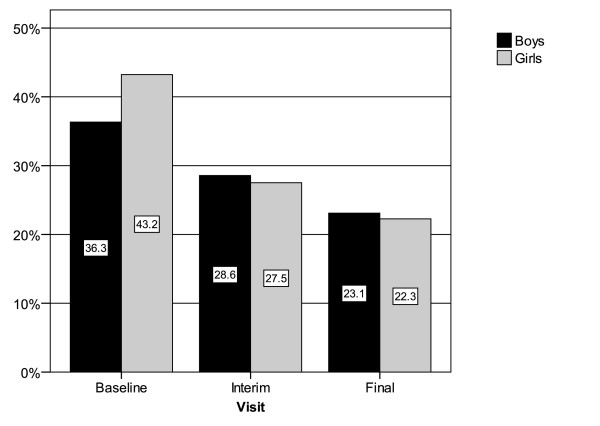
**Percentage of children with problems to fall asleep at the three different interviews**. Boys: 567; girls: 229.

A gender-specific evaluation revealed that at baseline girls had more problems with falling asleep (43.2% of n = 229 vs. 36.3% of n = 567). During the course of the study this difference disappeared, indicating that with regard to "falling asleep" girls drew more benefit from taking ESPRICO^® ^than boys (Figure 5).

### Assessment of safety and tolerability

At the final interview tolerability and safety of the food supplement was assessed by the investigators and the parents of the participating children. The observing physicians assessed the tolerability of PUFA in combination with magnesium and zinc to be very good or good in 711 children (87.8%), moderate in 45 children (5.6%), and poor in 11 children (1.4%). Likewise, the parents rated the tolerability as very good or good in 82.9% of the children, as moderate in 73 children (9.0%), and as poor in 20 children (2.5%). At the end of the study period the observers recommended continuation of intake in 495 (61.1%) of the participating children.

#### Adverse events

A total of 31 adverse events (AEs) were reported by 24 children (3.0% of 810) during the observational period of 12 weeks. All AEs were considered by the investigators to be mild or moderate in intensity, and no serious adverse event (SAE) occurred. According to the investigators, 16 AEs reported by 14 children (1.7%) had a possible, probable or confirmed causal relationship to the intake of the food supplement. The most frequently reported AEs were reactions of the gastrointestinal tract, e.g. nausea or vomiting, and psychiatric disorders (Table 6). Nine children (1.1%) discontinued the intake of the dietary supplement in the context of an intolerance reaction. All patients experiencing AEs recovered without sequels.

**Table 6:** Adverse events classified by the observers to have a possible, probable or confirmed causal relationship to the consumption of ESPRICO^® ^(MedDRA System Organ Classes and Preferred Terms).

	N	%
Infections and parasitic diseases	1	0.1

Infection	1	0.1

Psychiatric diseases	5	0.6

Difficulty sleeping through the night	1	0.1

Restlessness	1	0.1

Sleep-related problems	2	0.2

Behavioural disorder	1	0.1

Diseases of the gastrointestinal tract	8	1.0

Abdominal symptoms	1	0.1

Distended abdomen	1	0.1

Abdominal pain	2	0.2

Dyspepsia	1	0.1

Nausea	1	0.1

Vomiting	2	0.2

Diseases of the skin and hypodermis	1	0.1

Urticaria	1	0.1

Injury, intoxication and complications resulting from interventional procedures	1	0.1

Nausea in connection with a procedure	1	0.1

Children with no adverse reactions	796	98.3

Children, total	810	100

### Assessment of acceptance

At the final interview the acceptance of ESPRICO^® ^consumption was assessed by the parents (Table 7). According to these ratings ESPRICO^® ^was always, or at least usually, taken willingly by 526 children (64.9%). A total of 60 children (7.4%) refused to take the dietary supplement at least occasionally. An analysis of different age groups revealed that children between 4 and 7 years of age refused (always or sometimes) much more often (21 of 153; 13.8%) than older children (39 of 612; 6.4%).

**Table 7:** Acceptance of ESPRICO^® ^according to the parents.

**Children **...	N	%
... always took it willingly	226	27.9%

... usually took it willingly	300	37.0%

... sometimes took it only after "verbal encouragement"	179	22.1%

... sometimes refused	44	5.4%

... always refused	16	2.0%

No information	45	5.6%

Total	810	100%

## Discussion

### ADHD and Emotions

Most subjects showed a substantial reduction in symptoms of attention deficit and hyperactivity/impulsivity as well as of emotional and behavioural problems. These findings are in line with many other studies reporting a positive effect of PUFA on ADHD symptoms [32-35]. Also micronutrients like magnesium [36,37] and particularly zinc have proved to be important with regard to treatment of ADHD [22,38] and improvement of ADHD related symptoms [39,40].

### Sleep

Sleeping disorders, mainly problems to fall asleep, decreased significantly during the 12 week consumption of PUFA in combination with magnesium and zinc, with girls having more benefit than boys and older children having more problems to fall asleep. These findings could be related to emotional problems, which are known to be correlated with sleep problems: The calmer and more balanced a person is, the better the sleep quality; the better the sleep quality, the better daytime attention and concentration will be. This inter-relation is probably also the reason for the gender-specific differences: Girls showed higher SDQ-scores for emotional problems over the whole study period and accordingly they had more problems to fall asleep; the same explanation applies for older children. An underlying biochemical mode of action involves the polyunsaturated fatty acids (PUFA), which are structural and functional components of cell membranes and pre-stages of the hormonally and immunologically active eicosanoids. These eicosanoids play an important role in neural function, including sleep induction via prostaglandin D2 [41]. Lavialle et al. [42] fed hamsters a PUFA-deficient diet and found a 52% reduction in the nocturnal peak level of melatonin compared to control hamsters (P = 0.001). In accordance with Scheer and Czeisler [43] they conclude that a PUFA-deficient diet may play a role in nocturnal sleep disturbances via melatonin release also in humans.

### Tolerability

Overall, the safety profile of the food supplement is characterized by a low rate of side effects. The participating children or their parents reported neither severe nor serious adverse events, and the majority of potentially related events subsided spontaneously. A total of 16 adverse events with a possible causal relationship to the study medication were reported by 14 children (1.7%). Nine children (1.1%) discontinued the study because of incompatibility reactions. Most frequently, subjects experienced reactions of the gastrointestinal tract (Table 6), e.g. nausea or vomiting, which can be considered as common intolerance reactions to preparations containing fish oils [32]. Nausea and burps are also common after a regular fish meal [44]. To our knowledge there are no reports of any serious reactions after consumption of PUFA. A study using nutritional supplements comparable to this specified combination of PUFA, magnesium and zinc did not find any physiologically relevant changes (blood cell count, liver enzymes) in the verum group, too [45].

### Acceptance

Acceptance problems with consumption of the food supplement occurred rarely. However, a total of 60 children (7.4%) did not take the supplement at least occasionally and only 42 children (5.2%) discontinued participation in the study due to acceptance problems. Thus, this nutritional supplement was accepted by a large majority of the study population ensuring a sufficient level of compliance.

### Limitations

This observational, longitudinal study is subject to the limitations applying to this type of study in general. For this reason, the level of evidence is lower as compared with randomized controlled trials or with case-control studies. Due to the lack of a control group and no control of treatment allocation, multiple confounding cannot be ruled out. However, due to the large number of participants and the longitudinal design, this study can serve as a basis for further controlled studies. In detail, several limitations have to be addressed: The validity of the compliance reports is limited since data could not be cross-validated by pill counts or other methods of compliance control. Therefore compliance may have been overestimated. Furthermore, the study situation itself may have contributed to increased attention paid to the children during the observational period, influencing the outcome of the study. Also we did not control for socioeconomic status, which might influences effectiveness [39]. Additionally, comparisons with other effectiveness studies are limited by methodology, e.g., by differences in the scales used as outcome measures. Eligibility for participation was based on the presence of symptoms of inattention and behavioural problems rather than on a confirmed ADHD diagnosis and co-morbidities. Nevertheless, to date this is the largest observational study in this area, assessing more than 800 subjects in a longitudinal design.

## Conclusion

Due to the common nutritional practices in industrialized western countries, with high amounts of processed food products containing mostly very low levels of long-chained omega-3 fatty acids (DHA and EPA), one can expect a discrepancy between desirable and actual intake of these long-chained omega-3 fatty acids in both adults and children. But DHA and EPA can also be synthesized from alpha-linolenic acid (ALA), an omega-3 fatty acid, in the liver through a series of elongation and de-saturation steps. ALA itself occurs in a number of green vegetables as well as in certain nuts and seeds. However, there have been recent concerns that the efficiency of the conversion process may be low because both omega-3 and omega-6 fatty acids share and compete for the same enzymes that are used for de-saturation and elongation [46]. Omega-6 fatty acids, such as linoleic acid (LA) and arachidonic acid (AA), are widely present in vegetable oils, seeds, nuts, margarine, grains, eggs, and meats, whereas PUFAs are found primarily in canola and soybean oil, flaxseed, walnuts, eggs, some meats, and cold water fish (2). Highest concentrations of DHA and EPA are found only in very few fatty cold water fish, e.g., salmon, mackerel and herring. Thus, intake of omega-6 fatty acids is much higher than that of omega-3 fatty acids which may be associated with an increased risk of mental health disorders [7,47] and other diseases like arteriosclerosis [48].

New strategies for improving this poor dietary situation in the general population are needed considering the fact that fish stocks are limited and that mercury poisoning might be dangerous when eating high amounts of cold water fish (mainly shark, swordfish, king mackerel, tuna and tilefish). Various authorities have already given adequate recommendations. They include food supplements such as fish oil capsules or functional foods like microencapsulated omega-3 fatty acid products. For example, eggs, yogurt, milk, and spreads have been enriched with these fatty acids [44].

Taking both the essential nutritional role of omega-3 fatty acids EPA and DHA on attention and concentration in children [overviews in [6] and [49]] and the favourable risk-benefit ratio of the preparation into account, the results of this study support a recommendation of the use of PUFA in combination with magnesium and zinc in children. Similar suggestions for supplementation of PUFA have been made before [50].

With regard to children diagnosed to suffer from ADHD this study may be of heuristic importance, but it cannot establish a proof of efficacy due to its methodological limitations. However, taking concerns of the parents, teachers and patients regarding stimulant treatment into account, it is important to propel insights into innovative approaches to help children and adolescents with ADHD. PUFA do not necessarily contradict the therapy; however, if efficacy can be proven by controlled studies, it may serve as a primary and supportive approach. Adequate dosage of PUFA for ADHD-patients needs to be examined for they seem to have a different fatty acid metabolism [C51, 52].

## Competing interests

The authors declare that they have no competing interests and that this manuscript is original, has not been published before and is not currently being considered for publication elsewhere. The conduct of the study and preparation of this manuscript was supported by an educational grant from Engelhard Arzneimittel GmbH & Co KG, Niederdorfelden, Germany. Prof. Huss collaborates with Engelhard Arzneimittel in the development of dietary therapy in ADHD. Data analysis and interpretation were not influenced by the company.

## Authors' contributions

All authors contributed equally to this manuscript. The authors listed confirm that the manuscript has been read and approved by all named authors and that there are no other persons who satisfied the criteria for authorship but are not listed. The authors further confirm that the order of authors listed in the manuscript has been approved by all authors.
